# D-Chiro-Inositol – Its Functional Role in Insulin Action and its Deficit in Insulin Resistance

**DOI:** 10.1080/15604280212528

**Published:** 2002

**Authors:** Joseph Larner

**Affiliations:** 1 Department of Pharmacology University of Virginia School of Medicine Charlottesville Virginia 22908 USA; 2 Insmed Inc., PO Box 2400 Glen Allen, VA 23058 USA

## Abstract

In this review we discuss the biological significance of D-chiro-inositol, originally discovered as a component of a putative mediator of intracellular insulin action, where as a putative mediator, it accelerates the dephosphorylation of glycogen synthase and pyruvate dehydrogenase, rate limiting enzymes of non-oxidative and oxidative glucose disposal.

Early studies demonstrated a linear relationship between its decreased urinary excretion and the degree of insulin resistance present. When tissue contents, including muscle, of type 2 diabetic subjects were assayed, they demonstrated a more general body deficiency. Administration of D-chiro-inositol to diabetic rats, Rhesus monkeys and now to humans accelerated glucose disposal and sensitized insulin action.

A defect *in vivo* in the epimerization of myoinositol to chiro-inositol in insulin sensitive tissues of the GK type 2 diabetic rat has been elucidated. Thus, administered D-chiro-inositol may act to bypass a defective normal epimerization of myo-inositol to D-chiro-inositol associated with insulin resistance and act to at least partially restore insulin sensitivity and glucose disposal.


*Int. Jnl. Experimental Diab. Res.*, 3:47-60, 2002
© 2002 Taylor and Francis
1560-4284/02 $12.00 + .00

### D-Chiro-Inositol – Its Functional Role in Insulin Action and Its Deficit in Insulin Resistance

JOSEPH LARNER, M.D., PH.D.*

Department of Pharmacology, University of Virginia School of Medicine, Charlottesville, Virginia 22908, USA

**In this review we discuss the biological significance of D-chiro-inositol, originally discovered as a component of a putative mediator of intracellular insulin action, where as a putative mediator, it accelerates the dephosphorylation of glycogen synthase and pyruvate dehydrogenase, rate limiting enzymes of non-oxidative and oxidative glucose disposal.**

**Early studies demonstrated a linear relationship between its decreased urinary excretion and the degree of insulin resistance present. When tissue contents, including muscle, of type 2 diabetic subjects were assayed, they demonstrated a more general body deficiency. Administration of D-chiro-inositol to diabetic rats, Rhesus monkeys and now to humans accelerated glucose disposal and sensitized insulin action.**

**A defect *in vivo* in the epimerization of myo-inositol to chiro-inositol in insulin sensitive tissues of the GK type 2 diabetic rat has been elucidated. Thus, administered D-chiro-inositol may act to bypass a defective normal epimerization of myo-inositol to D-chiro-inositol associated with insulin resistance and act to at least partially restore insulin sensitivity and glucose disposal.**

*Keywords*: D-chiro-inositol, insulin resistance,diabetes, myo-inositol, epimerase,phosphoprotein phosphatase,insulin sensitizer,GK rat,glucose disposal

### BACKGROUND AND INTRODUCTION

The stereoisomeric family of 9 inositols includes myo-, cis-, allo-, epi-, muco-, neo-,

*Corresponding author: email: jlld@virginia.edu; current address: Insmed Inc., PO Box 2400, Glen Allen, VA 23058, USA; e-mail: jlarner@insmed.com

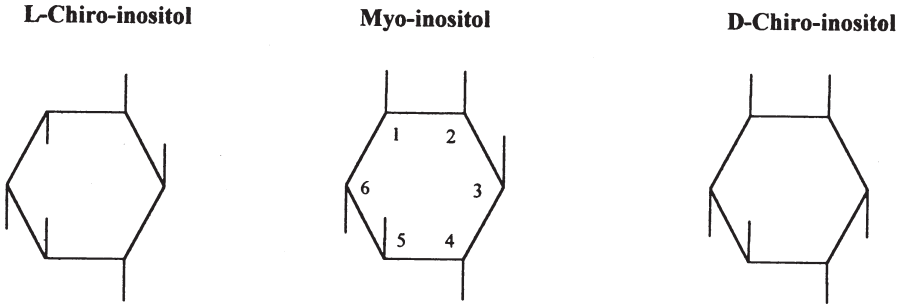

FIGURE 1
Structures of myo-, D and L chiro-inositols. Note that myo-inositol is epimerized in position 1 to form L-chiro-inositol and in position 3 to form D-chiro-inositol.

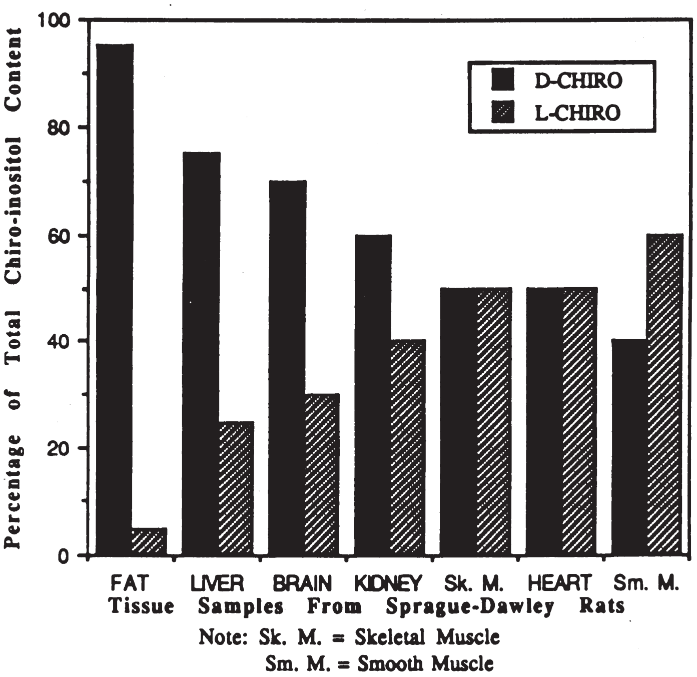

FIGURE 2
Tissue distribution of rat phospholipid D and L chiro-inositols.
Tissues were extracted in methanolic chloroform. Extracts were dried *in vacuo* and hydrolyzed in 6N Hcl. Analysis of inositols was by GC/MS as hepta fluorobutyryl esters. From Ph.D. Thesis, Allison S. Kennington, University of Virginia (1990) p. 65 "Insulin Mediators and Their Inositol Contents". Note the predominance of D-chiro-inositol in fat, liver, brain and kidney phospholipids and the approximately equal amounts of D and L chiro-inositol in skeletal muscle, heart and smooth muscle.

scyllo- and the optical isomers D and L chiro-inositols. Myo-inositol is the most widely distributed in nature. Biosynthesized from glucose [1], the cyclase converting the immediate precursor fructose 6-P to myo-inositol has been cloned [2]. L-chiro-inositol is the product of epimerizing hydroxyl #1 of myo-inositol, while D-chiro-inositol is the product of epimerizing hydroxyl #3 of myo-inositol (Fig. 1). D-chiro-inositol has classically been found in plants and insects. A rich source in plants is pinitol, the 3-0-methyl ether extracted from pine wood [3]. L-chiro-inositol is also found in plants and parasites as quebrachitol, its 2-0-methyl ether [4]. L-chiro-inositol is also found in beef liver and rat tissue phospholipids [5, 6].

Early reports noted the presence of minor amounts of chiro-inositol in animal and human tissue sources including rat granulation tissue [7], human placenta, human urine and uremic serum [8] in the presence of much greater quantities of myo-inositol. Another report identified chiro-inositol together with myo-inositol in a mixed glycosyl phosphatidyl inositol lipid (GPI) fraction from rat liver and H35 hepatoma cells, without determining the absolute configuration of the chiro-inositol [9].

Two separate inositol phosphoglycan (IPG) putative mediators of insulin action from rat liver were purified and chemically analyzed in our lab. The one which activated pyruvate dehydrogenase phosphatase (PDH) contained D-chiro-inositol and galactosamine, the second which inhibited cAMP kinase and adenylate cyclase contained myo-inositol and glucosamine [10]. This was the first definitive demonstration of the sole presence of D-chiro-inositol in an IPG molecule without myo-inositol being present. In this review, we shall summarize the current status of the biological significance of D-chiro-inositol in animals, its functional role in insulin action, its deficit as related to insulin resistance, and current studies of its epimerization from myo-inositol.

### BIOLOGICAL SIGNIFICANCE OF CHIRO-INOSITOL AND ITS IPG

### *Tissue Distribution of Chiro-inositol*

An early study demonstrated the presence of both D and L chiro-inositol in rat tissues (Fig. 2)

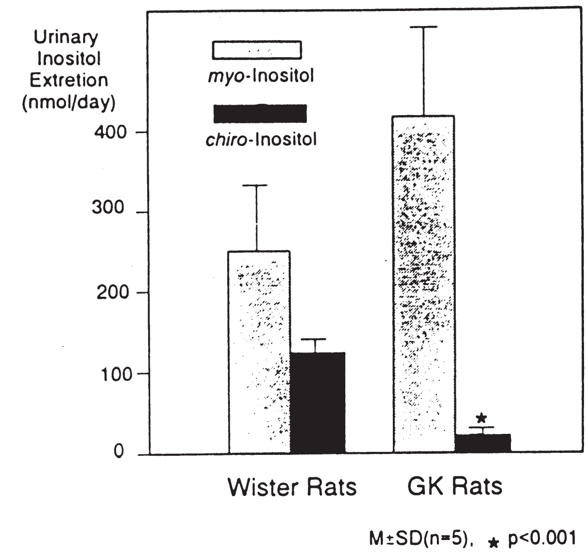

FIGURE 3
24 hr urinary excretion of myo- and chiro-inositols in G/K rats. Inositols were analyzed by GC/MS as described by Kennington, *et al.* (Ref. 11) From Ref. 14 with permission. Note the similar pattern of excretion as seen in Rhesus monkeys (Table 1) of increased myo-inositol and decreased chiro-inositol.

TABLE 1
24 hr urinary excretion of myo and chiro-inositols in Rhesus monkeys.
Urine was collected in the presence of toluene and sodium azide in iced bottles to minimize bacterial contamination. Note the pattern of decreased urinary chiro-inositol and increased urinary myo-inositol as the animals progressed from normal to obese non-diabetic to type 2 diabetic monkeys. From [11] with permission.

| Monkey no. | Diagnosis | *chiro*-Inositol (μmol/day) | *myo*-Inositol (μmol/day) |
|---|---|---|---|
| 1 | Normal | 18.5 | 21.9 |
| 2 | Normal | 3.1 | 5.9 |
| 3 | Normal | 1.8 | 6.7 |
| 4 | Normal | 3.7 | 3.5 |
| Mean ± SE | — | 6.7 ± 2.3 | 9.5 ± 2.4 |
| 5 | Obese | 1.7 | 7.0 |
| 6 | Obese | 3.6 | 6.5 |
| 7 | Obese | 2.9 | 11.3 |
| 8 | Obese | 0.3 | 3.1 |
| 9 | Obese | ND | 72.2 |
| 10 | Obese | ND | 63.2 |
| 11 | Obese | ND | 4.9 |
| Mean ± SE | — | 1.2 ± 0.2 | 24.0 ± 4.7 |
| 12 | Diabetic | 1.0 | 169.0 |
| 13 | Diabetic | ND | 61.4 |
| 14 | Diabetic | ND | 312.5 |
| 15 | Diabetic | ND | 61.3 |
| Mean ± SE | — | 0.2 ± 0.1 | 151.1 ± 34.3 |

ND, not detectable (limit of detection <0.5 μmol/l). Adapted from Kennington et al. (69).

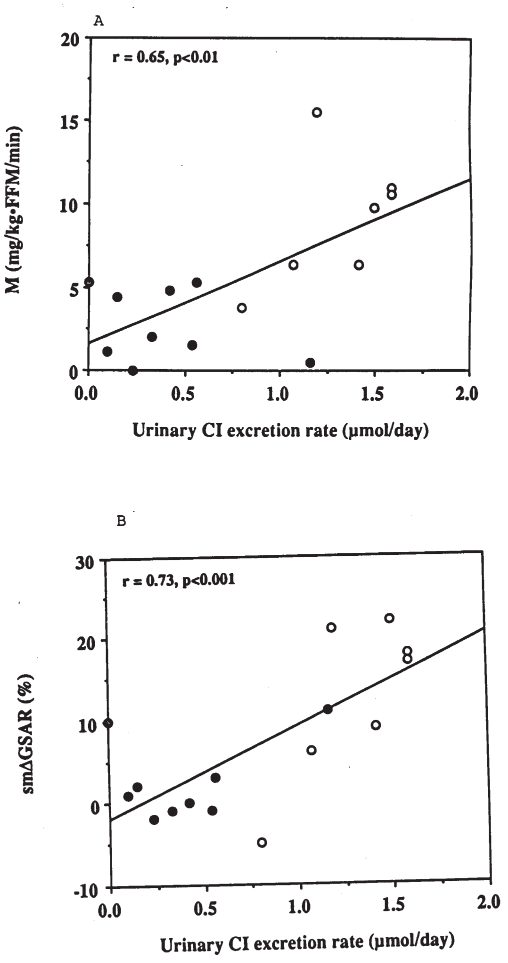

FIGURE 4
24 hr urinary chiro-inositol excretion rate and insulin sensitivity. Normal monkeys •; NIDDM monkeys •; hyperinsulinemic monkeys •
Insulin sensitivity determined by the hyperinsulinemic euglycemic clamp procedure.
Insulin sensitivity determined by skeletal muscle biopsy glycogen synthase activation state. Note the relationship between 24 hr excretion rate of chiro-inositol and insulin resistance.
From [15] with permission.

[6]. In crude phospholipids extracted from fat, liver, brain and kidney the D-isomer predominated whereas in skeletal muscle, heart and smooth muscle approximately equal amounts of D and L isomers were present. The amounts present were much smaller than myo-inositol. In rat urine, D-chiro-inositol again predominated over the L-isomer as it did in human urine as well [11,12]. This demonstrated that both D and L isomers have a widespread distribution in rat tissues and that the D isomer predominated in specific tissues and in urine.

### *Urinary Excretion of Chiro-Inositol-Relationship to Diabetes*

To understand the functional significance of the two inositol containing IPG putative insulin mediator species, we initially examined the 24 hr urinary myo and chiro-inositol excretions in human subjects. We had much earlier demonstrated increased urinary myo-inositol excretion in type 2 diabetic subjects compared with controls utilizing a yeast growth bioassay and shown that the increased excretion was due to a competition between glucose and myo-inositol in renal tubular transport [13]. Following our discovery of D-chiro-inositol in one IPG and myo-inositol in a second, we were interested to examine their roles particularly in insulin action. We therefore measured their urinary excretion in control and type 2 diabetic subjects using a modern GC/MS analysis. We reconfirmed the increased myo-inositol excretion in type 2 diabetics compared to controls, but to our surprise we also noted a decreased excretion of chiro-inositol [11]. This was noted in a local cohort of subjects at Virginia and in a population of Pima Indians as well. A similar pattern was also observed in the rhesus monkey (Table 1) where an increase of myo-inositol and a decrease of chiro-inositol excretion was observed in the progression from normal, to obese non-diabetic, to type 2 diabetic [11]. This pattern of increased myo-inositol and decreased chiro-inositol excretion in urine has also been reported in GK rats, a non-obese type 2 diabetic model developed in Japan by inbreeding rats selectively for insulin resistance in [14] (Fig. 3).

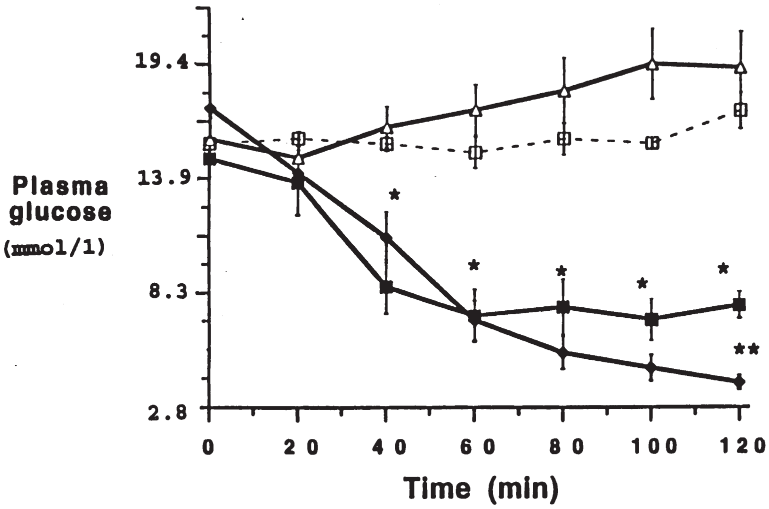

FIGURE 5
Effects on plasma glucose of infusions of 150 mmol/L NaCl (Δ N=7); insulin 73 p mol/min (• N=6); IPG-P 8.5 pmol/min (low) (• N=3); and IPG-P 83.3 pmol/min (high) (• N=5) Data are mean ± SEM; *$p<0.001$ **$p<0.05$. From (Ref. 21) with permission. Note the lack of hypoglycemia in the 2nd hour of infusion with IPG-P compared to insulin.

### *Urinary Excretion of Chiro-Inositol-Relationship to Insulin Resistance.*

That the decreased urine chiro-inositol was more strictly related to insulin resistance per se, rather than to type 2 diabetes was next shown in rhesus monkeys [15]. Insulin sensitivity determined by the euglycemic hyperinsulinemic glucose clamp M value, glucose disappearance rate (Kglucose), muscle biopsy glycogen synthase activation and phosphorylase inactivation states, and adipose tissue glycogen synthase activation and phosphorylase inactivation states all correlated with urinary chiro-inositol excretions [15]. Two examples are shown in Fig. 4: the correlation of urinary chiro-inositol excretion with M values (Fig. 4A) and with skeletal muscle glycogen synthase activation state (Fig. 4B) [15]. As seen, decreased urine chiro-inositol excretion rate is related linearly to decreased insulin sensitivity (increased insulin resistance). Similar correlations have now been obtained in humans comparing controls with glucose intolerant non-diabetics and type 2 diabetics [16]. Thus, it has become clear that decreased urine chiro-inositol as well as increased myo-inositol may be measures of insulin resistance. Altered ratios of increased myo-inositol to decreased chiro-inositol in urine have been proposed as a more sensitive index of insulin resistance in human subjects [17]. A recent independent/confirmation by Wall *et al.* has also appeared in abstract form [18]. These and other early studies in which urine and tissue (muscle) inositols [19] were examined indicated that a generalized total body deficiency of chiro-inositol was associated with tissue resistance to insulin action.

### INSULIN LIKE *IN VIVO* ACTIONS OF IPG'S AND D-CHIRO-INOSITOL

### *In Vivo and In Vitro IPG Actions*

The two separate purified IPG's from rat liver, one containing D-chiro-inositol and

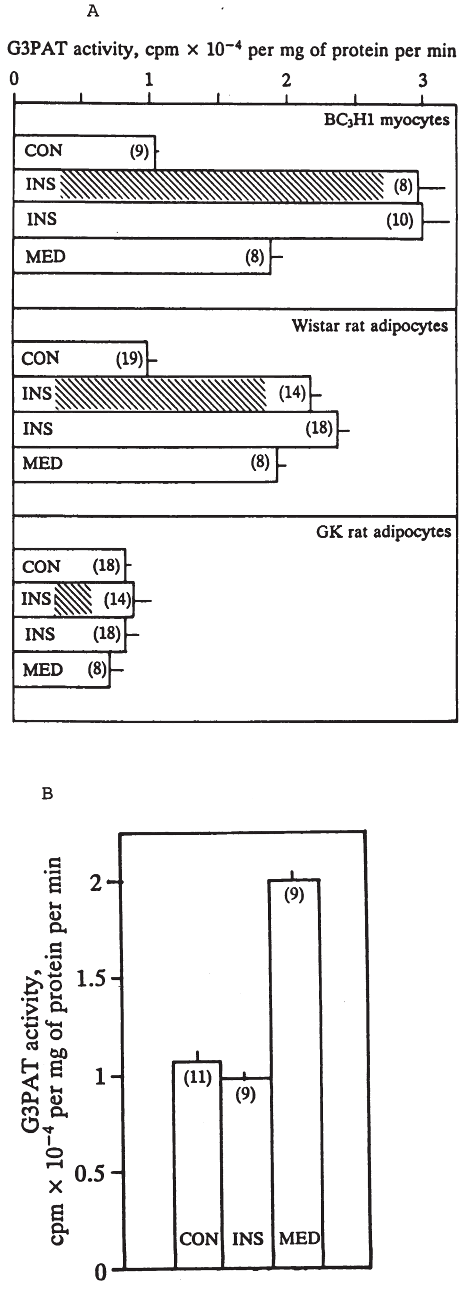

FIGURE 6
A. Effects of insulin (INS) or IPG-P (MED) on G3PAT activity (CPM x $10^{-4}$ mg prot/min) in intact (hatched bars) or cell free homogenates (open bars) of $BC_3H_1$ myocytes (top) non-diabetic Wistar rat adipocytes (middle) and diabetic G/K rat adipocytes (bottom). Values are means ± SE of (n) assays. Note the lack of insulin and IPG-P action in the G/K rat adipocytes compared to the controls.

B. Effects of insulin (INS) and IPG-P (Med) on G3PAT activation in cell free homogenates of insulin-treated diabetic G/K rats (2u NPH insulin injected for 6 days prior to experiment). Note the activating effect of IPG-P in the absence of an effect of insulin. From [23] with permission.

termed IPG-P (inositol phosphoglycan-phosphatase stimulator) and the other containing myo-inositol and termed IPG-A (inositol phosphoglycan-AMP kinase inhibitor) were then injected into low-dose STZ rats, a model of type 2 diabetes. They were shown to reduce hyperglycemia in a dose-dependent manner [20]. Single bolus IV injections of low nanomolar doses reduced hyperglycemia about 50%. Subsequently, using an infusion protocol in the same animal model, an approximately equivalent low nanomolar dose of infused insulin was compared with a similar dose of IPG-P [21]. Both effectively reduced hyperglycemia to euglycemia during the first 60 min, but in the second 60 min period, insulin produced a hypoglycemic response, while the IPG-P maintained euglycemia (Fig. 5). Two control infusions with saline and low (1/10) dose of IPG-P are also shown. While we do not as yet understand this difference between IPG-P and insulin action, IPG-P in contrast to IPG-A has been shown in preliminary experiments to specifically inhibit glucose stimulated insulin release from isolated rat islet beta cells [22] thus constituting a potential feedback mechanism. Since these IPG's are clearly insulin-like *in vivo* in reducing hyperglycemia at doses comparable to insulin, this evidence is in keeping with their potential roles as insulin mediators with IPG-P perhaps somewhat more physiological in its action than IPG-A.

Further evidence for a more selective role of the IPG-P derives from an experiment demonstrating its insulin like action in GK rat broken cell preparations. In both $BC_3H_1$ myocytes and in adipocytes from Wistar rats, insulin and IPG-P stimulated insulin sensitive glycerol 3 phosphate acyltransferase (G3PAT) activity comparably in whole cells and homogenates while both were ineffective in GK rat adipocytes (Fig. 6A) [23]. However, if G/K rats were pretreated with insulin for 6 days, presumably to restore protein phosphatase activi-

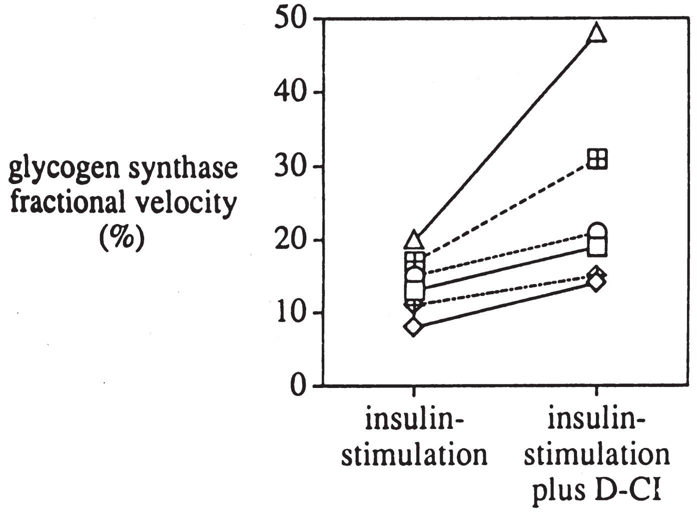

FIGURE 7
Skeletal muscle glycogen synthase fractional velocity under insulin stimulated (euglycemic hyperinsulinemic clamp) and insulin-stimulated plus D-chiro-inositol in 6 monkeys. D-chiro-inositol was administered I.V. 1g/K for 3 min. 30 min later the last muscle biopsy was performed. At steady state maximally effective insulin infusion rates, D-chiro-inositol further activated glycogen synthase above maximal insulin levels. From [30] with permission.

ty, strikingly, adipocyte homogenates from GK rats became sensitive to G3PAT stimulation by IPG-P under conditions where insulin was still ineffective possibly due to a chiro-inositol synthesis deficit (See Epimerization) or an IPG synthesis or IPG- generation deficit (Fig. 6B) [23]. Since IPG-A was inactive in these experiments, this again is a striking demonstration of the insulin-like effectiveness of IPG-P in a diabetic resistant tissue where insulin itself was still ineffective, again evidence for the IPG-P acting as a potential insulin mediator.

Of interest, Schofeld and Hackett also demonstrated that myo-inositol GPI from P. falciparum also had insulin-like effects *in vitro* and *in vivo* [24]. Acting on adipocytes it increased, [$^{14}$C] glucose oxidation to [$^{14}CO_2$] and [$^{14}$C] lipid synthesis. In mice, IP injection of 12.5 µg GPI lowered blood glucose and caused hypoglycemia not attributable to TNF release. Thus the myo-inositol GPI lipid was insulin mimetic. Unfortunately the IPG was not tested in these *in vitro* and *in vivo* assays for its insulin mimetic properties.

Although somewhat counter intuitive to classical second messenger theory, there is now a solid body of data supporting the extracellular generation/release of IPG's. These include extracellular antibody inhibition studies [25], cultured cell dilution experiments demonstrating a predicted decreasing effect of increased culture medium volume on bioactivity [26] and impermeant flourescent tag experiments [27] all supporting the unconventional thesis. In keeping with an extracellular site of generation, an ATP-dependent transport for IPG has been reported in rat hepatocytes [28] thus demonstrating a mechanism for cell entry from extracellular sites.

### *In Vivo D-Chiro-inositol Action*

Since urine and tissue concentrations of chiro-inositol and putative mediator levels were decreased in type 2 diabetes with insulin resistance [6, 11,19], we reasoned that D-chiro-inositol administration might replete IPG putative

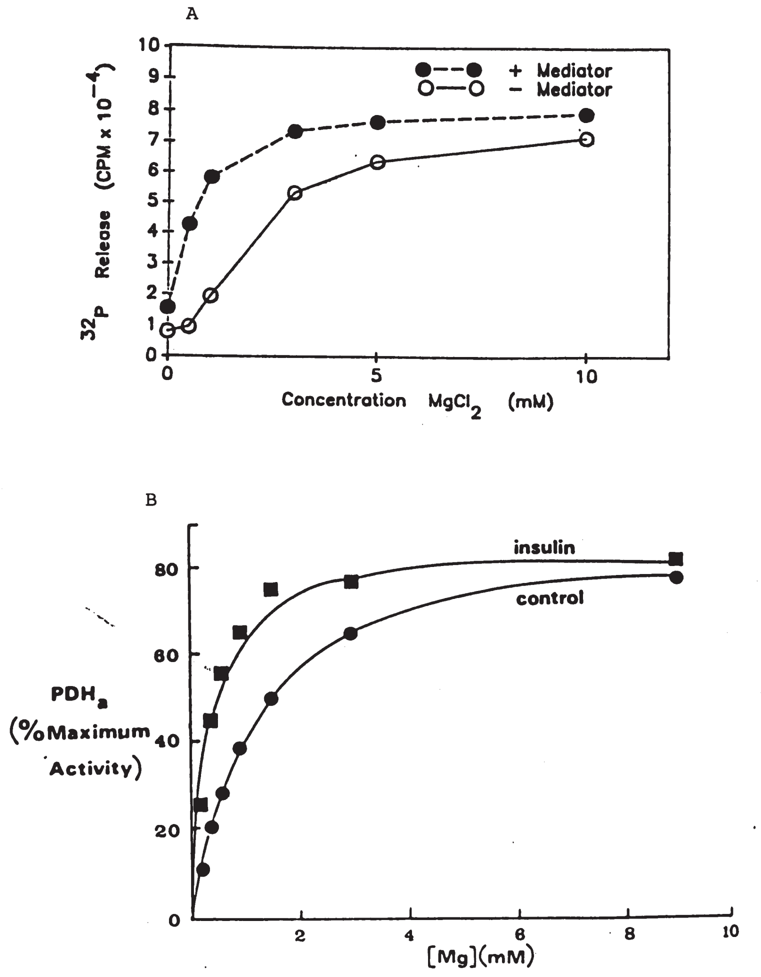

Figure 8

A. Effect of $MgCl_2$ on the stimulatory effect of IPG-P putative mediator. The reaction mixture contained homogenous PDH phosphatase (supplied by Dr. L. Reed) and [$^{32}$P] PDH complex. Compare the left shifted $MgCl_2$ sensitivity in the presence of IPG-P with the action of insulin shown in B. From (Ref. 36) with permission.

B. Effect of insulin on the sensitivity of pyruvate dehydrogenase phosphatase to $Mg^{2+}$ . Toluene permeabilized mitochondria from control (•) and insulin-treated (•) tissue were incubated and steady state values of pyruvate dehydrogenase complex activity (PDHa) were obtained in the presence of 0.2 mM ATP, 100 µM $Ca^{2+}$, and various $Mg^{2+}$ concentrations. From [39] with permission.

mediator stores. In contrast to IPG which is administered parenterally, D-chiro-inositol may be administered orally. Initial studies in low-dose STZ rats demonstrated that in the dose range of 1-10 mg/kg, a dose dependent modest decrease in hyperglycemia of up to 50% was observed. Similarly when administered I.v. to diabetic and insulin resistant rhesus monkeys (100 mg/kg), increased rates of decline of plasma glucose and plasma insulin were noted [29]. A striking demonstration of the effect of D-chiro-inositol to enhance insulin sensitivity was an experiment in which the activation state of glycogen synthase was determined in muscle biopsies during a euglycemic hyperinsulinemic clamp in the Rhesus monkey. While insulin

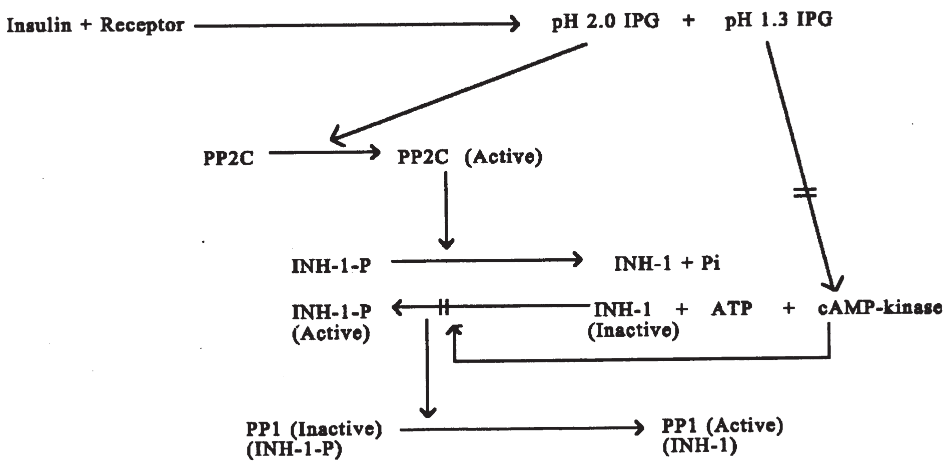

FIGURE 9
Model of serial activation of PP1 and PP2C by IPG-P and IPG-A. Dephosphorylation of INH-1-P is accomplished by both IPG-P and IPG-A. IPG-P stimulates PP2C to dephosphorylate while IPG-A inhibits its phosphorylation by PKA. Thus, PP1 becomes activated by virtue of the loss of inhibitory activity of INH-1. Similar experiments were also, conducted with DARPP-32. From [43] with permission.

alone increased GS activation state (Fig. 7, not shown) the further infusion of D-chiro-inositol in the continued presence of maximal insulin further activated the enzyme above that produced by maximal insulin (Fig. 7) [30]. Presumably the D-chiro-inositol is acting by initial incorporation into GPI lipid precursor stores, with subsequent release by phospholipase action as IPG-P stimulated by insulin. We have previously identified and characterized the presence of chiro-inositol containing phospholipids in bovine liver [31]. More recently, we have shown that [$^3$H] D-chiro-inositol injected *in vivo* is incorporated into control Wistar and diabetic G/K rat phospholipids [32]. Insulin stimulated release of IPG from GPI lipids is well established [23].

In another model of insulin resistance, glucosamine was infused into rats for 210 min to induce insulin resistance under euglycemic glucose clamp conditions [33]. Animals pretreated with either troglitazone or D-chiro-inositol for 7 days were compared. Pretreatment with D-chiro-inositol had no effect on hepatic glucose output but prevented glucosamine induced peripheral insulin resistance, whereas pretreatment with troglitazone improved hepatic glucose output but was without effect on peripheral insulin resistance. This again indicates the effectiveness of D-chiro-inositol to correct insulin resistance in an additional model of insulin resistance [33].

In separate experiments, we were unable to demonstrate any metabolic effects of myo-inositol administered under similar conditions to D-chiro-inositol [29]. However, Ortmeyer in rhesus monkeys was able to observe the improvement of glucose tolerance by myo-inositol. [34]. Diabetic conditions and/or animal species may produce these differences. A possible explanation raised by these latter authors was that the effectiveness of myo-inositol may have resulted from its conversion to chiro-inositol *in vivo*. This is addressed in a later section. It is of interest that Ostlund *et al.* [35] have demonstrated a $Na^+$ dependent transport of D-chiro-inositol in HepG2 liver cells by a transporter that also transports myo-inositol.

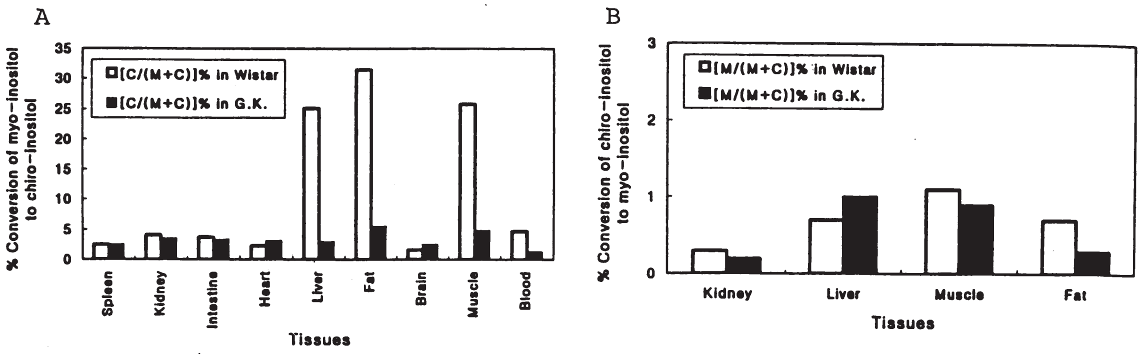

FIGURE 10
A. [$^3$H]myo-inositol conversion to [$^3$H]chiro-inositol in purified phospholipids of control Wistar and diabetic G/K tissues. % conversion was based on ratio of chiro-inositol/myo-inositol + chiro-inositol. Note the marked decrease in conversion of myo to chiro-inositol in the insulin sensitive tissues liver, fat and muscle.

B. [$^3$H]chiro-inositol conversion to [$^3$H]myo-inositol in the purified free inositols from control Wistar and diabetic G/K rat tissues. The % conversion was based on the ratio of myo-inositol/chiro-inositol + myo-inositol. Note the small back conversion of chiro to myo-inositol in all tissues. From (Ref. 51) with permission.

### *IN VITRO* INTRACELLULAR MECHANISM OF ACTION OF IPG'S

IPG-P is termed P since it activates phosphoprotein phosphatases. Originally found to activate PDH phosphatase [36], it was subsequently found to activate phosphatase PP2C as well [37]. Both PDH phosphatase and PP2C are now recognized as members of the family of phosphatases termed PPM (phosphoprotein phosphatase-metal activated), since they are both $Mg^{++}$ requiring. Recently PP2C's structure has been determined by x-ray crystallography and shown to contain in its active center a bimetallic ion pair involved in catalysis [38].

We initially demonstrated that IPG-P activates PDH type 1 phosphatase holoenzyme by left shifting the $Mg^{++}$ dose response curve thus sensitizing the enzyme to $Mg^{++}$ (Fig. 8A) [36]. Of considerable physiologically interest, Denton *et al.* [39] independently demonstrated that when rat adipose tissue segments treated with insulin are homogenized and examined for the $Mg^{++}$ sensitivity of PDH phosphatase, the curve is left-shifted in the insulin-pretreated compared with the control tissue (Fig. 8B). Note that in both A and B the greatest increase in $Mg^{++}$ sensitivity occurs at low physiological $Mg^{++}$ concentrations. This again argues that IPG-P is a putative mediator since in a defined *in vitro* enzyme system it reproduces the kinetic action of insulin acting on a sensitive tissue, (fat), to activate a sensitive enzyme, PDH phosphatase. We have also shown that IPG-P activates PP2C dephosphorylating myosin light chains by left shifting the $Mg^{++}$ dose response curve [37].

Interestingly, Ortmeyer *et al.* demonstrated that following insulin administration *in vivo* to rhesus monkeys, liver biopsy samples exhibited activation of *both* PP1 and PP2C [40]. Other investigators have also observed activation of PP1 in fat or liver cells treated with insulin [41,42]. Our laboratory demonstrated a mechanism for the serial activation of both PP2C and PP1 by the 2 IPG's, IPG-P and IPG-A in an *in vitro* model system [43]. As shown in the model (Fig. 9) both IPG putative mediators lead to decreased phosphorylation of INH1, a selective inhibitor of PP1 catalytic subunit.

IPG-P stimulates PP2C to dephosphorylate INH1 dose dependently in low nanomolar doses, while IPG-A inhibits cAMP-kinase mediated phosphorylation of INH1 dose dependently in similar low nanomolar doses [43]. This "double barrel" IPG putative mediator action disinhibits and thus activates PP1 following the preceding activation of PP2C by IPG-P. In keeping with this model, decreased phosphorylation of INH1 with *in vivo* insulin action has been noted in the literature [44].

IPG-A on the other hand inhibits both cAMP-kinase and adenylate cyclase [45] and thus helps explain the effect of insulin to reverse the action of counter regulatory hormones that elevate cAMP. Thus, both IPG's are insulin-like in their action *in vivo* [20] but, as already discussed, IPG-P seems more direct and specific in its role as a putative insulin mediator, while IPG-A may mediate the counter regulatory effect of insulin on epinephrine and glucagon.

### EPIMERIZATION OF MYO-INOSITOL TO CHIRO-INOSITOL

Early studies in Chlorella fusca [46] and the cockroach fat body [47] demonstrated the presence of NAD and NADH dependent inositol epimerase(s) interconverting myo-inositol into scyllo, chiro, and neo-inositols. The mechanism proposed was via initial oxidation of the inositol to a monoketone inosose by an oxidase, with NAD as electron and proton acceptor. This was followed by stereospecific reduction by a reductase to the epimeric inositol with NADH or NADPH as electron and proton donor. Using extracts from bovine brain, Hipps *et al.* [48] demonstrated the interconversion of myo- into scyllo- and neo-inositol. Chiro-inositol was not observed. Evidence for the presence of two separate enzymes was presented, an oxidase producing the inosose and a stereospecific reductase to form the epimeric inositol.

Our initial experiments demonstrated that when rats were injected with 1 mC of [$^3$H] myo-inositol over a 3 1/3 day period to approach isotopic steady state and the tissues examined for the presence of [$^3$H] chiro-inositol, the percent conversion of myo-to chiro-inositol was greatest in liver, muscle (insulin sensitive tissues) and blood (~8-9%). Significant but lower percent of conversion was observed in other tissues ranging from ~1 to 4% [49]. When the phospholipids were examined the percent conversion in blood was ~60%, in muscle ~8% and liver ~2% [49].

In a subsequent experiment, rat fibroblasts expressing the human insulin receptor were prelabeled with [$^3$H] myo-inositol for 48h and examined for the conversion to [$^3$H] chiro-inositol in the absence and presence of insulin. Insulin in 15 min stimulated the conversion of [$^3$H] myo-inositol phospholipid into [$^3$H] chiro-inositol phospholipid [50]. This result suggested that the epimerization stimulated by insulin occurred at the phospholipid level rather than at the free inositol level. Alternatively, it might have occurred at the free inositol level but with the rapid incorporation of the free inositol into phospholipids [32].

With the availability of [$^3$H] D-chiro-inositol, we next studied its overall tissue disposition in normal Wistar and GK diabetic rats together with its conversion to [$^3$H] myo-inositol. We also compared the overall distribution of [$^3$H] myo-inositol and its conversion to [$^3$H] chiro-inositol. Both labeled inositols were injected at a dose of 1 mC over a 3 1/3 day period, animals sacrificed, organs collected and analyzed for both [$^3$H] myo-inositol and [$^3$H] chiro-inositol [32]. When the percent conversion of [$^3$H]myo-inositol to [$^3$H] chiro-inositol was examined (Fig. 10) a marked decrease was observed in liver, fat, muscle (insulin sensitive tissues) and blood of the GK compared with Wistar control rats. As seen in Fig. 10A, in liver a decrease from ~25% (Wistar) to ~3% (GK), in fat a decrease from ~30% (Wistar) to ~6% (G/K), and in muscle a decrease from ~25% (Wistar) to ~5% (GK) was observed [32]. Of interest, only a small back conversion of [$^3$H]chiro-inositol to [$^3$H] myo-inositol was observed (Fig. 10B) (1% or less) with no significant difference between control and G/K rats. This suggests that *in vivo* the forward conversion of myo to chiro-inositol is essentially unidirectional. The data further indicates a significant defect in the conversion of myo- to chiro-inositol in the tissues (principally insulin sensitive tissues) of the G/K compared to the control Wistar rat. If such a defect in fact exists, it may help explain why administered chiro-inositol is effective in alleviating insulin resistance related to its deficiency.

### SUMMARY AND CONCLUSIONS

We have reviewed the growing body of evidence for the biological significance of D-chiro-inositol, its potential role when present in IPG-P as a putative mediator of insulin action. The decrease of chiro-inositol in urine was correlated with insulin resistance and its formation via epimerization from myo-inositol *in vivo*. Another recent review has summarized the role of insulin receptor activated alternate G-protein linked signaling mechanisms in the generation of IPG's [51]. Other reviews have dealt with more general aspects of IPG's and their role as putative insulin mediators [52] and as related to diabetes [53] as well as their more general role in hormone and growth factor signal transduction [54]. A chapter by Hansen and Ortmeyer reviewed experiments in the rhesus monkey dealing with insulin resistance and type 2 diabetes [55]. Clearly this is a rapidly growing scientific area of great theoretical and practical interest.

*ACKNOWLEDGEMENTS*

Generous support for this work was provided by a private donor, by a loan from the University of Virginia Patent Foundation, research grants from Insmed Pharmaceuticals, and the Center for Innovative Technology Commonwealth of Virginia.

### REFERENCES

1. Daughaday, W.H., Larner, J, and Hartnett, C. (1955) The synthesis of inositol in the immature rat and chick embryo. *J. Biol. Chem.* **212**, 869-875.
2. Dean-Johnson, M., and Henry, S.A. (1989) Biosynthesis of inositol in yeast. Primary structure of myo-inositol-1-phosphate synthase (EC5.5.1.4) and functional analysis of its structural gene, the IN01 locus. *J. Biol. Chem.* **264**, 1274-1283.
3. Anderson, A.B. (1953) Pinitol from sugar pine stump wood. *Ind. and Eng. Chem.* **45**, 593-596.
4. Schmatz, D.M., Arison, B.H., Dashkevicz, M.P., Liesch, J.M., Turner, M.J. (1988) Identification and possible role of D-mannitol and 2-0-methyl-chiro-inositol (quebrachitol) in Eimeria tenella. *Molec. and Biochem. Parasitol.* **29**, 29-36.
5. Bruzik, K.S., Hakeem, A.A., and Tsai, M.D. (1994) Are D- and L- chiro-phosphoinositides substrates of phosphoinositol-specific phospholipase. *C. Biochem.* **33**, 8367-8374.
6. Kennington, A.S. (1990) Insulin mediators and their inositol components. Ph.D. Thesis, University of Virginia.
7. Williamson, J.R., Chang, K., Rowold, E., Marvel, J., Tomlinson, M., Sherman, W.R. et al. (1986) Diabetes-induced increases in vascular permeability and changes in granulation tissue levels of sorbitol, myo-inositol, chiro-inositol, and scyllo-inositol are prevented by sorbinil. *Metabolism* **35**: (Suppl 1), 41-45.
8. Niwa, T., Yamamoto, N., Maeda, K., Yamada, K. (1983) Gas chromatographic-mass spectro-metric analysis of polyols in urine and serum of uremic patients. Identification of new deoxyalditols and inositol isomers. *J. Chrom.* **277**, 25-39.
9. Mato, J.M., Kelly, K.L., Abler, A., Jarett, L., Corkey, B.E., Cashel, J.A. et al. (1987) Partial structure of an insulin-sensitive glycophospholipid. *Biochem Biophys. Res. Commun.* **146**, 764-772.
10. Larner, J., Huang, L.C., Schwartz, C.F.W., Oswald, A.S., Shen, T.Y., Kinter, M., et al. (1988) Rat liver insulin mediator which stimulates pyruvate dehydrogenase phosphatase contains galactosamine and D-chiro-inositol. Biochem. *Biophys. Res. Commun.* **151**, 1416-1426.
11. Kennington, A.S., Hill, C.R., Craig, J., Bogardus, C., Raz, I., Ortmeyer, H.K., et al. (1990) Low urinary chiro-inositol excretion in noninsulin-dependent diabetes mellitus. *N. Eng. J. Med.* **323**, 373-378.

12. Ostlund, R.E., McGill, J.B., Herskowitz, I., Kipnis, D.M., Santiago, J.V., Sherman, W.R. (1993) D-chiro-inositol metabolism in diabetes mellitus. *Proc. Natl. Acad. Sci. USA* **90**, 9988-9992.

13. Daughaday, W.H., and Larner, J. (1954) The renal excretion of inositol in normal and diabetic human beings. *J. Clin. Invest.* **33**, 326-332.

14. Suzuki, S., Taneda, Y., Hirai, S., Abe, S., Sasaki, A., Suzuki, K., and Toyota, T. (1991) Molecular mechanism of insulin resistance in spontaneous diabetic GK (Goto-Kakizaki) rats. In New Directions in Research and Clinical Works for Obesity and Diabetes Mellitus. Angel. A., Hotta, N. eds. Elsevier, 197-203.

15. Ortmeyer, H.K., Bodkin, N.L., Lilley, K., Larner, J. and Hansen, B.C. (1993) Chiro-inositol deficiency and insulin resistance. I. Urinary excretion rate of chiro-inositol is directly associated with insulin resistance in spontaneously diabetic rhesus monkeys. *Endocrinology* **132**, 640-645.

16. Suzuki, S., Kawasaki, H., Satoh, Y., Ohtomo, M., Hirai, M., Hirai, A., Hirai, S., Onoda, M., Matsumoto, M., Hinokio, Y., Akai, H., Craig, J., Larner, J., and Toyota, T. (1994) Urinary chiro-inositol excretion is an index marker of insulin sensitivity in Japanese type II diabetes. *Diabetes Care* **17**, 1465-1468.

17. Larner, J. and Craig, J.W. (1996) Urinary myo-inositol-to-chiro-inositol ratios and insulin resistance. *Diabetes Care* **19**, (1) 76-78.

18. Wahl, H.G., Grener, B., Werner, R., Schmuelling, R.M. (1998) Urinary myo-/chiro-inositol ratio is increased in NIDDM patients and in non-diabetic first degree relatives. *Diabetes* **47**, A429 (Abstract).

19. Asplin, I., Galasko, G., and Larner, J. (1993) Chiro-inositol deficiency and insulin resistance: a comparison of the chiro-inositol and the myo-inositol containing insulin mediators isolated from urine, hemodialysate and muscle of control and type II diabetic subjects. *Proc. Natl. Acad. Sci. USA* **90**, 5924-5928.

20. Huang, L.C., Fonteles, M.C., Houston, D.B., Zhang, C., and Larner, J. (1993) Chiro-inositol deficiency and insulin resistance. III. Acute glycogenic and hypoglycemic effects of two inositol phosphoglycan insulin mediators in normal and streptozotocin diabetic rats in vivo. *Endocrinology* **132**, 652-657.

21. Fonteles, M.C., Huang, L.C., and Larner, J. (1996) Infusion of pH 2.0 D-chiro-inositol glycan insulin putative mediator normalizes plasma glucose in streptozotocin diabetic rats at a dose equivalent to insulin without inducing hypoglycemia. *Diabetologia* **39**, 731-734.

22. Egan, J.M., Asplin, C.M., Evans, W.S., Huang, L.C., and Larner, J. (1991) The effect of a chiro-inositol containing insulin mediator on insulin release in rats (Abstract). *Diabetes* **40**, (Suppl. 1) 187A.

23. Farese, R.V., Standaert, M.L., Yamada, K., Huang, L.C., Zhang, C., Cooper, D.R., Wang, Z., Tang, Y., Suzuki, S., Toyota, T. and Larner, J. (1994) Insulin-induced activation of glycerol-3-phosphate acyltransferase by a chiro-inositol-containing insulin mediator is defective in adipocytes of insulin-resistant, type II diabetic, Goto-Kakizaki rats. *Proc. Natl. Acad. Sci. USA* **91**, 11040-11044.

24. Schofield, L. and Hackett, F. (1993) Signal transduction in host cells by a glycosyl phosphatidyl inositol toxin of malaria parasites. *J. Exp. Med.* **177**, 145-153.

25. Romero, G., Gamez, G., Huang, L.C., Lilley, K., and Luttrell, L. (1990) Anti-inositol glycan antibodies selectively block some of the actions of insulin in intact $BC_3H1$ cells. *Proc. Natl. Acad. Sci. USA* **87**, 1476-1480.

26. Romero, G., Garmey, J.C., and Veldhuis, J. (1993) The involvement of inositol phosphoglycan mediators in the modulation of steroidogenesis by insulin-like growth factor-1. *Endocrinology* **132**, 1561-1568.

27. Variela, I., Alvarez, J.F., Clemente, R., Ruiz-Albusac, J.M., and Mato, J.M. (1990) Asymmetric distribution of the phosphatidylinositol-linked phospho-oligosaccharide that mimics insulin action in the plasma membranes. *Eur. J. Biochem.* **188**, 213-218.

28. Alvarez, J.F., Sanchez-Arias, J.A., Guadano, A., Estevez, F., Varela, I., Feliu, J.E., and Mato, J.M. (1991) Transport in isolated rat hepatocytes of the phospho-oligosaccharide that mimics insulin action. Effects of adrenalectomy and glucocorticoid treatment. *Biochem. J.* **274**, 369-371.

29. Ortmeyer, H.K., Huang, L.C., Zhang, L., Hansen, B.C., and Larner, J. (1993) Chiro-inositol deficiency and insulin resistance. II. Acute effects of D-chiro-inositol administration in streptozotocin-diabetic rats, normal rats given a glucose load, and spontaneously insulin-resistant rhesus monkeys. *Endocrinology* **132**, 646-651.

30. Ortmeyer, H.K., Bodkin, N.L., Hansen, B.C., and Larner, J. (1995) In vivo D-chiro-inositol activates skeletal muscle glycogen synthase and inactivates glycogen phosphorylase in Rhesus monkeys. *J. Nutr. Biochem.* **6**, 499-503.

31. Pak, Y. and Larner, J. (1992) Identification and characterization of chiro-inositol-containing phospholipids from bovine liver. *Biochem. Biophys. Res. Commun.* **184**, 1042-1047.

32. Pak, Y., Hong, Y., Kim, S., Piccariello, T., Farese, R.V., and Larner, J. (1998) In vivo chiro-inositol metabolism in the rat: a defect in chiro-inositol synthesis from myo-inositol and an increased incorporation of chiro-[$^3$H]inositol into phospholipid in the Goto-Kakizaki (G.K.) *Rat. Mol. Cells* **8**, (3) 301-309.

33. Yoshino, Y., Takeda, N., Sugimoto, M., Nakashima, K., Okumura, S., Hattori, J., Sasaki, A., Kawachi, S., Takami, K., Takami, R., and Yasuda, K. (1999) Differential effects of troglitazone and D-chiro-inositol on glucosamine-induced insulin resistance in rats. *Metabolism* **48**, 1418-1423.

34. Ortmeyer, H.K. (1996) Dietary myo-inositol results in lower urine glucose and in lower post prandial plasma glucose in obese insulin resistant Rhesus monkeys. *Obesity Res.* **4**, 569-575.

35. Ostlund, R.E., Seemayer, R., Gupta, S., Kimmel, R., Ostlund, E.L., and Sherman, W.R. (1996) A stereospecific myo-inositol/D-chiro-inositol transporter in HepG2 liver cells: Identification with D-chiro[3-$^3$H]inositol. *J. Biol. Chem.* **271**, 10073-10078.

36. Larner, J., Huang, L.C., Suzuki, S., Tang, G., Zhang, C., Schwartz, C.F.W., Romero, G., Luttrell, L. and Kennington, A.S. (1989) Insulin mediators and the control of pyruvate dehydrogenase. *Ann. N.Y. Acad. Sci.* **573**, 297-305.

37. Abe, S., Huang, L.C., and Larner, J. (1996)

Dephosphorylation of PDH by phosphoprotein phosphatases and its allosteric regulation by inositol glycans. In alpha-keto-acid dehydrogenase complexes. Patel, M.S., Roche, T.E., Harris, R.A. Eds. Basel, Birkhauser, 187-195.

38. Das, A.K., Helps, N.R., Cohen, P.T.W., and Barford, D. (1996) Crystal structure of the protein serine/threonine phosphatase 2C at 2.0 Å resolution. *Embo J.* **15**, 6798-6809.

39. Denton, R.M., Midgley, P.J.W., Rutter, G.A., Thomas, A.P., and McCormack, J.G. (1989) Studies into the mechanism whereby insulin activates pyruvate dehydrogenase complex in adipose tissue. *Ann N.Y. Acad. Sci.* **573**, 285-296.

40. Ortmeyer, H.K. (1998) Insulin increases liver protein phosphatase 1 and protein phosphatase 2C activates in lean, young adult Rhesus monkeys. *Horm. Metab. Res.* **30**, 705-710.

41. Brady, M.J., Bourbonais, F.J., and Saltiel, A.R. (1998) The activation of glycogen synthase by insulin switches from kinase inhibition to phosphatase activation during adipogenesis in 3T3-L1 cells. *J. Biol. Chem.* **273**, 14063-14066.

42. Ragolia, L., and Begum, N. (1998) Protein phosphatase 1 and insulin action. *Mol.Cell. Biochem.* **182**, 49-58.

43. Huang, L.C., Heimark, D., Linko, J., Nolan, R., and Larner, J. (1999) A model phosphatase 2C phosphatase 1 activation cascade via dual control of inhibitor 1 (INH-1) and DARPP-32 dephosphorylation by two inositol glycan putative insulin mediators from beef liver. *Biochem. Biophys. Res. Commun.* **255**, 150-156.

44. Foulkes, J.G., Cohen, P., Strada, S.J., Everson, W.V., and Jefferson, L.S. (1982) Antagonistic effects of insulin and ß-adrenergic agonists on the activity of protein phosphatase inhibitor-1 in skeletal muscle of the perfused rat hemicorpus. *J. Biol. Chem.* **257**, 12493-12496.

45. Malchoff, C.D., Huang, L.C., Gillespie, N., Villar-Palasi, C., Schwarz, C.F.W., Cheng, K., Hewlett, E.L., and Larner, J. (1987) A putative mediator of insulin action which inhibits adenylate cyclase and adenosine 3', 5'-monophosphate-dependent protein kinase: partial purification from rat liver, site and kinetic mechanism of action. *Endocrinology* **120**, 1327-1337.

46. Wober, G., and Hoffman-Ostenhof, O. (1969) Untersuchungen uber die Biosynthese der Cyclite, 22 Mitt.: Cyclite in Chlorella fusca. *Monatsh. Chem.* **100**, 369-375.

47. Hipps, P.P., Sehgal, R.K., Holland, W.H. and Sherman, W.R. (1973) Identification and partial characterization of inositol $NAD^+$ epimerase and inosose: NAD(P)H reductase from the body fat of American cockroach. *Periplaneta Americana L. Biochemistry* **12**, 4705-4712.

48. Hipps, P.P., Ackermann, K.E. and Sherman, W.R. (1982) Inositol epimerase-Inosose reductase from bovine brain. *Meth. In Enzymol.* **89**, 593-598.

49. Pak, Y., Huang, L.C., Lilley, K.J., and Larner, J. (1992) In vivo conversion of [$^3$H]myo-inositol to [$^3$H]chiro-inositol in rat tissues. *J. Biol. Chem.* **267**, 16904-16910.

50. Pak, Y., Paule, C.R., Bao, Y-D., Huang, L.C., and Larner, J. (1993) Insulin stimulates the biosynthesis of chiro-inositol containing phospholipids in a rat fibroblast line expressing the human insulin receptor. *Proc. Natl. Acad, Sci. USA* **90**, 7759-7763.

51. Larner, J. and Huang, L.C. (1999) Identification of a novel inositol glycan signaling pathway with significant therapeutic relevance to insulin resistance: an insulin signaling model using both tyrosine kinase and G proteins. *Diabetes Rev.* **7**, 217-231.

52. Field, M.C. (1997) Is there evidence for phospho-oligosaccharides as insulin mediators. *Glycobiology* **7**, 161-168.

53. Jones, D.R. and Varela-Nieto, I. (1999) Diabetes and the role of inositol-containing lipids in insulin signaling. *Molec. Med.* **5**, 505-514.

54. Varela-Nieto, I., Leon, Y. and Caro, H.N. (1996) Cell signaling by inositol phosphoglycans from different species. *Comp. Biochem. Physiol.* **115B**, 223-241.

55. Hansen, B.C. and Ortmeyer, H.K. (1996) Inositols potential roles in insulin action and in diabetes: Evidence from insulin resistant nonhuman primates. Lessons from Animal Diabetes VI, E. Shafrir Ed. Birkhuser, Boston 333-348.

